# Rapid screening for specific glycosylation and pathogen interactions on a 78 species avian egg white glycoprotein microarray

**DOI:** 10.1038/s41598-017-06797-6

**Published:** 2017-07-25

**Authors:** Marta Utratna, Heidi Annuk, Jared Q. Gerlach, Yuan C. Lee, Marian Kane, Michelle Kilcoyne, Lokesh Joshi

**Affiliations:** 1 Glycoscience Group, National Centre for Biomedical Engineering Science, National University of Ireland Galway, Galway, Ireland; 2 Regenerative Medicine Institute, National University of Ireland Galway, Galway, Ireland; 3 Department of Biology, Johns Hopkins University, 3400 North Charles Street, Baltimore, Maryland 21218 USA; 4 Carbohydrate Signalling Group, Microbiology, School of Natural Sciences, National University of Ireland Galway, Galway, Ireland

## Abstract

There is an urgent need for discovery of novel antimicrobials and carbohydrate-based anti-adhesive strategies are desirable as they may not promote resistance. Discovery of novel anti-adhesive molecules from natural product libraries will require the use of a high throughput screening platform. Avian egg white (EW) provides nutrition for the embryo and protects against infection, with glycosylation responsible for binding certain pathogens. In this study, a microarray platform of 78 species of avian EWs was developed and profiled for glycosylation using a lectin panel with a wide range of carbohydrate specificities. The dominating linkages of sialic acid in EWs were determined for the first time using the lectins MAA and SNA-I. EW glycosylation similarity among the different orders of birds did not strictly depend on phylogenetic relationship. The interactions of five strains of bacterial pathogens, including *Escherichia coli*, *Staphylococcus aureus* and *Vibrio cholera*, identified a number of EWs as potential anti-adhesives, with some as strain- or species-specific. Of the two bacterial toxins examined, shiga-like toxin 1 subunit B bound to ten EWs with similar glycosylation more intensely than pigeon EW. This study provides a unique platform for high throughput screening of natural products for specific glycosylation and pathogen interactions. This platform may provide a useful platform in the future for discovery of anti-adhesives targeted for strain and species specificity.

## Introduction

With the rise of antimicrobial resistance in human pathogens and the serious consequences of untreatable infections for human healthcare, there is increased interest in sourcing novel antimicrobial molecules, especially those which do not promote resistance^1, 2^. The food industry in particular is interested in natural antimicrobial compounds that could be effective against food spoilage and pathogenic microorganism growth^3^. Adhesion of bacteria to the host cell surface is one of the first and most critical steps in infection and anti-adhesion therapy has been shown experimentally to be effective for preventing or treating infections^4, 5^. Most bacteria express cell surface adhesins that bind to particular host cell surface carbohydrates. Virus particles and bacterial toxins can also have carbohydrate-binding protein or lectin domains to bind to host cells. For example, the P-fimbriae of uropathogenic *Escherichia coli* and Shiga-like toxin 1 (Stx1) binds to the Gal-α-(1,4)-Gal (galabiose) structure found on human glycolipids in fibroblasts, erythrocytes, primary kidney cells, and intestinal tissue^6–8^. Thus binding inhibitor or anti-adhesive molecules that are structurally similar to the complex carbohydrate ligands presented by the host have been extensively investigated in recent years. Multivalent presentation of these anti-adhesives are typically more effective than the monovalent version and significantly lower concentrations of the multivalent anti-adhesives are required compared to the monovalent versions^4, 5, 9^. Therefore, discovery of novel anti-adhesive molecules, including those that are species- or even strain-specific, will require screening multivalently-presented carbohydrates with sufficient structural complexity to function as effective decoys or mimics of host carbohydrates and a high-throughput (HTP) screening method or platform^4, 5^.

Avian egg white (EW), or albumen, provides nourishment to the developing embryo and has been suggested to act as a defensive layer, shielding the embryo from pathogens. Chicken EW (CEW) comprises approximately 60% of the total egg weight and constitutes 10–12% of total protein^10^. Most EW proteins are glycoproteins, including ovalbumin (OVA), ovomucoid (OVM) and ovotransferrin (OVT) which are modified with *N*-linked oligosaccharides^11–14^. Protein glycosylation has important biological roles in many processes including mediating host-pathogen interactions, immune response, cell development and differentiation. EW is growth restricting for pathogens as it contains multiple antimicrobial components, including lysosyme (LYZ) and OVT^10, 15^. Additionally, the structures, linkages and presentation of oligosaccharides present on EW glycoproteins serve as ligands for microbial adhesion^10^, presumably to arrest bacterial migration to the yolk and facilitate subsequent antimicrobial defence. The Gal-α-(1,4)-Gal terminal structure is found in EW oligosaccharides of a few species of birds distributed in closely positioned branches of the phylogenetic tree including pigeon EW (PEW), in amphibians and in a sea turtle^16–18^. Thus EW glycoproteins may provide an excellent source of natural antimicrobials and specificity for targeted pathogens could potentially be selected for, depending on appropriate EW glycosylation.

Enzymatic glycosylation of proteins is controlled by factors that differ greatly among cell types and species^19^. Oligosaccharide structures from a small number of purified EW glycoproteins have been previously analysed by mass spectrometric methods, the majority of which are *N*-linked oligosaccharides^11, 12, 20–22^, with a small number of *O*-linked structures elucidated from CEW ovomucin^23, 24^. A variety of crude and purified EW glycoproteins from a range of avian species have also been studied using lectin and antibody blotting^16, 25^. Lectins, particularly those from plants, are used to detect and profile intact glycosylation on glycoconjugates, cells and tissues^26^. High mannose (Man) and hybrid *N*-linked structures dominate in OVA while other CEW glycoproteins contain complex *N*-linked oligosaccharides with up to five antennae^12^. Pigeon glycoproteins contain up to four Gal-α-(1,4)-Gal and α-(2,6)-linked *N*-acetylneuraminic acid (Neu5Ac) residues on the termini of the antennae of their *N*-linked oligosaccharides^20, 21^. While it is important to characterize the individual EW glycoprotein oligosaccharides, the density, distribution pattern, and three-dimensional presentation of the intact oligosaccharides on the molecule greatly impact their biological interactions and recognition^19^. Accordingly, screening for EW glycosylation and interactions with targeted pathogens should be performed with EW in its natural conformation and distribution.

While lectin and glycan microarrays are well known and widely used for profiling glycosylation and carbohydrate-based interactions, the use of microarrays comprised of other glycosylated molecules are more recent developments. A natural human milk oligosaccharide microarray was developed which was utilized for screening of biologically relevant interactions^27^. A microarray of mucins purified from the reproductive and gastrointestinal tracts of six animals and from two cell lines was an effective tool for profiling mucin glycosylation^19^ and the interactions of intact bacterial pathogens^28^. No multiplexed presentation of a collection of EWs has been performed to date. The presentation of EWs in a HTP platform such as a microarray will be highly advantageous for understanding EW natural glycosylation and screening for interactions with targeted pathogens and their toxins while using minimal amounts of samples and analytes.

In this work, a natural avian EW microarray was constructed using EWs from 78 different species (Table S-1). Lectin profiling was carried out to characterise the EW glycosylation and deduced structures were correlated with previous studies and with avian phylogeny. As sialic acid linkages can be a critical factor in dictating pathogen binding to host ligands^29^, the linkages of sialic acids in EW species were elucidated in this study for the first time using *Maackia amurensis* agglutinin (MAA) and *Sambucus nigra* agglutinin I (SNA-I) lectins, which bind specifically to α-(2,3)- and α-(2,6)-linked sialic acid, respectively. The novel EW microarray was also used to investigate interactions with five bacterial strains of relevance to food, including *E*. *coli*, *Staphylococcus aureus* and the emerging foodborne pathogen *Vibrio parahaemolyticus*, and two bacterial toxins. This platform may be useful in the future for screening for novel species- and strain-specific potential anti-adhesives, which may be suitable for future deployment in food and food processing.

## Results and Discussion

### Electrophoretic analysis of avian EW library

**Figure 1:**
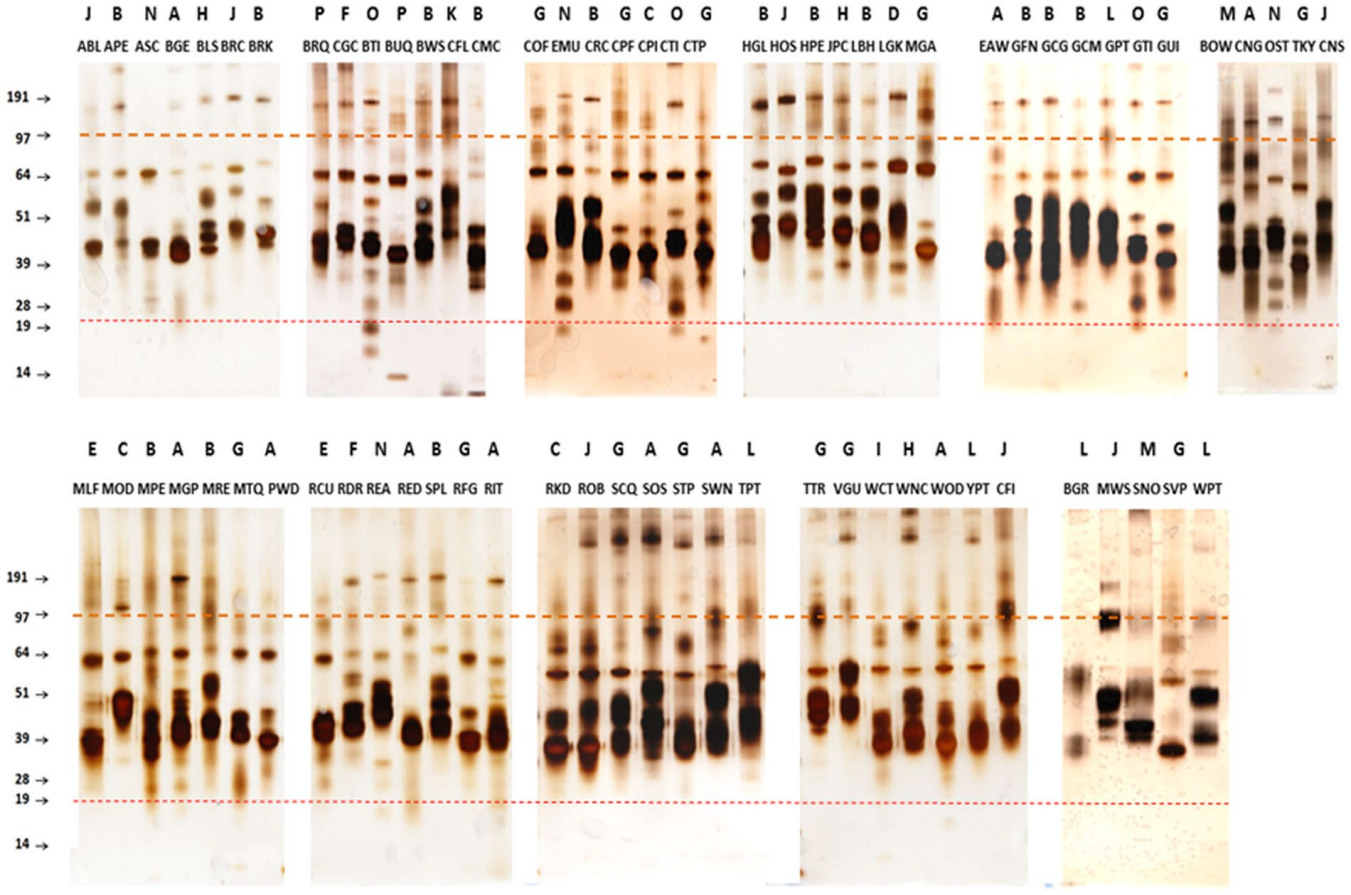
Electrophoretic profiles of EWs (with exception of CEW, DEW, GEW, PEW and QEW). EWs (1–3 μg per lane) were separated on NuPage 4–12% Bis-Tris gels and silver stained. The single capital letters above the gels illustrate the systematic order of birds while triple letters indicate abbreviated common names of birds (Table S-1). In each gel, 5 μL of molecular mass marker and 0.5 μg of purified chicken (CH) protein standards (OVT, OVA, OVM and LYZ) were separated (not shown for simplification). For comparison, the gels were aligned based on the migration of two marker bands: 97 (orange line) and 19 kDa (red line).

Following optimization of EW solubilisation (see Supplementary Information), the entire EW library was profiled by SDS-PAGE. The major protein components of CEW are OVA (45 kDa, 54% of total protein), OVT (77 kDa, 12%), and OVM (28 kDa, 11%)^10^ and these glycoproteins were used as references for electrophoretic analysis of all species of EWs (Fig. S-1A). OVA, OVT and OVM were evident in CEW and the majority of the other 77 species in the selected EW library (Table S-1 and Fig. 1), with different electrophoretic mobilities observed between species, in agreement with a previous study^16^. These mobility variations were previously attributed to differences in the amino acid sequences of individual glycoproteins and species-specific differences in the number and size of *N*-linked glycans in a comparison between CEW and pigeon EW (PEW)^21^.

Similar electrophoretic patterns for OVA, OVM and OVT for species in close phylogenetic proximity were previously observed for pheasant (SVP) and ostrich (OST) EWs in addition to quail EW (QEW), duck EW (DEW) and CEW^30^. However, in the present study, which contained a larger library of species, a corresponding species relationship was not conclusively demonstrated by electrophoretic analysis. Several characteristics observed in quantitative protein distribution in the various EWs were in agreement with previous studies. For example, the major protein component in emu EW (EMU) was OVT not OVA as for CEW and the electrophoretic mobilities of OVA and OVM varied in EMU compared to CEW (Fig. 1)^15^.

Interestingly, a 14 kDa band corresponding to LYZ (approximately 3.5% of total CEW protein)^10^ was observed in CEW and as a very faint band in DEW and QEW (Figs S-1A and 1) and small buttonquail EW (BUQ). However, no other EW in the library displayed a band with similar electrophoretic mobility to LYZ. This is due to low abundance or lack of LYZ in the EW of most bird species, e.g. only 1% of DEW is LYZ, 0.5% of goose and no LYZ has been found in EMU, or dramatically different electrophoretic mobility of the LYZ in other species^30^.

### Total CEW and individual CEW glycoprotein glycosylation

**Figure 2:**
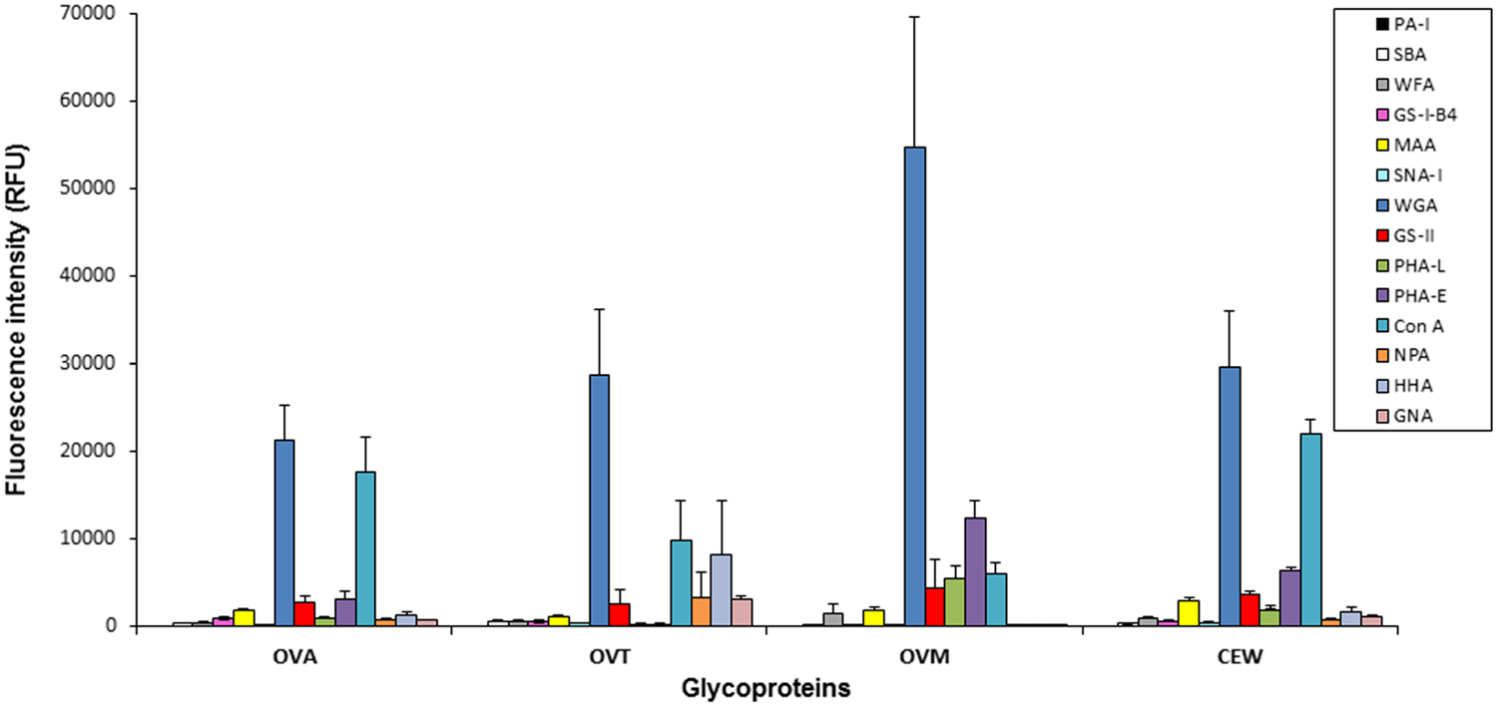
Bar chart representing the mean fluorescence intensity from three replicate experiments of 14 individual lectins with carbohydrate-specific interactions binding to three chicken EW glycoproteins (OVA, OVT and OVM) and total CEW on the EW microarray. Error bars are one standard deviation for the mean of all replicates (Table S-3).

Following optimization of microarray printing (Supplementary Fig. S1), the EW microarrays were constructed and incubated with a library of fluorescently-labelled lectins (Table 1) to generate a characteristic glycoprofile for each EW and standard. Appropriate haptenic carbohydrates or glycoproteins were also co-incubated with lectins to confirm that the lectin interactions with printed EWs were carbohydrate-mediated^26, 31^ (Table S-3). Initially individual lectin binding profiles for the purified CEW glycoproteins were examined for similarity to the glycoprofile for crude CEW (Fig. 2).

**Table 1:** Lectins used, their abbreviation, source species, binding specificity, concentration used and their haptenic sugars (100 mM) or glycoproteins (5 mg/mL).

Abbrev.	Conc. (µg/ml)	Source	Species	Specificity	Hapten
PA-I	0.1	Bacteria	*Pseudomonas aeruginosa*	Gal, Gal derivatives	Gal
AIA (Jacalin)	15	Plant	*Artocarpus integrifolia*	Gal (somewhat sialylation tolerant)	Gal
SBA	15	Plant	*Glycine max*	GalNAc	Gal
WFA	10	Plant	*Wisteria floribunda*	GalNAc/sulfated GalNAc	Gal
VVA-B4	10	Plant	*Vicia villosa*	GalNAc	Gal
PNA	15	Plant	*Arachis hypogaea*	Gal-β-(1→3)-GalNAc	Gal
VRA	20	Plant	*Vigna radiata*	Terminal α-linked Gal	Gal
GS-I-B4	4	Plant	*Griffonia simplicifolia* (*Bandeiraea simplicifolia*)	Terminal α-linked Gal	Gal
Con A	5	Plant	*Canavalia ensiformis*	α-linked Man, Glc or GlcNAc	Man
NPA	20	Plant	*Narcissus pseudonarcissus*	α-(1→6)-linked Man	Man
HHA	5	Plant	*Hippeastrum hybrid*	Man-α-(1→3)-Man-α-(1→6)-R	Man
GNA	4	Plant	*Galanthus nivalis*	Man-α-(1→3)-R	Man
MAA	10	Plant	*Maackia amurensis*	Neu-α-(2→3)-Gal(NAc)-R	Lac
SNA-I	2	Plant	*Sambucus nigra*	Neu-α-(2→6)-Gal(NAc)-R	Lac
WGA	0.04	Plant	*Triticum vulgaris*	NeuAc/GlcNAc	GlcNAc
GSL-II	15	Plant	*Griffonia simplicifolia*	GlcNAc	GlcNAc
PHA-L	2	Plant	*Phaseolus vulgaris*	Tri-, tetra-antennary β-Gal/Gal-β-(1→4)-GlcNAc	α-1-acid glycoprotein
PHA-E	0.7	Plant	*Phaseolus vulgaris*	Biantennary, bisecting GlcNAc, β-Gal/Gal-β-(1→4)-GlcNAc	Bovine IgG
UEA-I	15	Plant	*Ulex europaeus*	Fuc-α-(1→2)-R	Fuc

Of the purified CEW components, OVM had the highest binding intensity for the majority of the lectin panel, possibly due to its high carbohydrate content of 25% (w/w)^10^. In comparison, OVA and OVT contain 3.5% and 2.6% (w/w) carbohydrate, respectively^32, 33^. Although high binding intensity with LYZ was observed for most of the lectins (Table S-3), these interactions were not inhibited by haptenic sugars and were thus considered non-carbohydrate mediated. Interestingly, LYZ hydrolyses the linkage between the *N*-acetylglucosamine (GlcNAc) and *N*-acetylmuramic acid in the bacterial cell wall. All three CEW glycoproteins (OVA, OVT and OVM) interacted with the lectins wheat germ agglutinin (WGA, *Triticum vulgaris* agglutinin) and *Griffonia simplicifolia* lectin II (GSL-II), which indicated the presence of GlcNAc residues (Table 1). Only OVT interacted with the mannose-(Man-)specific lectins (*Narcissus pseudonarcissus* agglutinin (NPA), *Hippeastrum hybrid* agglutinin (HHA), and *Galanthus nivalis* agglutinin (GNA)) which indicated the presence of high-mannose type *N*-linked structures^32^. NPA and HHA bind internal and terminal Man residues with preference towards high Man type *N*-linked structures containing α-(1,6)-linked Man or both α-(1,3)- and α-(1,6)-linked Man, respectively (Table 1). GNA is reported to bind most strongly to multiple terminal α-(1,3)-linked Man^34^. Interaction with Concanavalin A (Con A, *Canavalia ensiformis* agglutinin), which has high binding affinity for terminal Man and Man core structures of *N*-linked glycans, was observed for all three glycoproteins. HHA binding was low with crude CEW, but moderate with OVT. This may be due to the OVT oligosaccharides comprising a relatively low proportion of crude CEW sample. However, the binding intensity of Con A was higher for crude CEW than the individual CEW glycoproteins which may reflect the overall larger relative proportion of high mannose type *N*-linked glycosylation.

The presence of bi-, tri- and tetra-antennary complex *N*-linked oligosaccharides, terminating most often with type II *N*-acetyllactosamine (LacNAc; Gal-β-(1,4)-GlcNAc) which is often sialylated^34^, on OVM was suggested by binding of both *Phaseolus vulgaris* erythroagglutinin (PHA-E) and *Phaseolus vulgaris* leucoagglutinin (PHA-L), while OVA only interacted with PHA-E and OVT did not bind with either lectin (Table 1 and Fig. 2). Furthermore, the presence of α-(2,3)-linked sialic acid on OVM was indicated by MAA binding, in agreement with previous reports^13, 33^. Also, WGA binding, which recognises GlcNAc and sialic acid (Table 1), was observed only for OVM and OVT. The results for OVT and OVM correlated well with structures previously elucidated by MS and HPLC^13^. OVT *N*-linked oligosaccharides were mainly composed of GlcNAc (54%) and Man (43%) and some Gal residues (3.5%), while the structures reported for OVM were mostly complex-type, with dominating tri-, tetra- and penta-antennary structures^13^. Overall, individual lectin binding profiles were observed for each purified CEW glycoproteins with a similar combination glycoprofile observed for crude CEW.

### EW library glycosylation profiling

**Figure 3:**
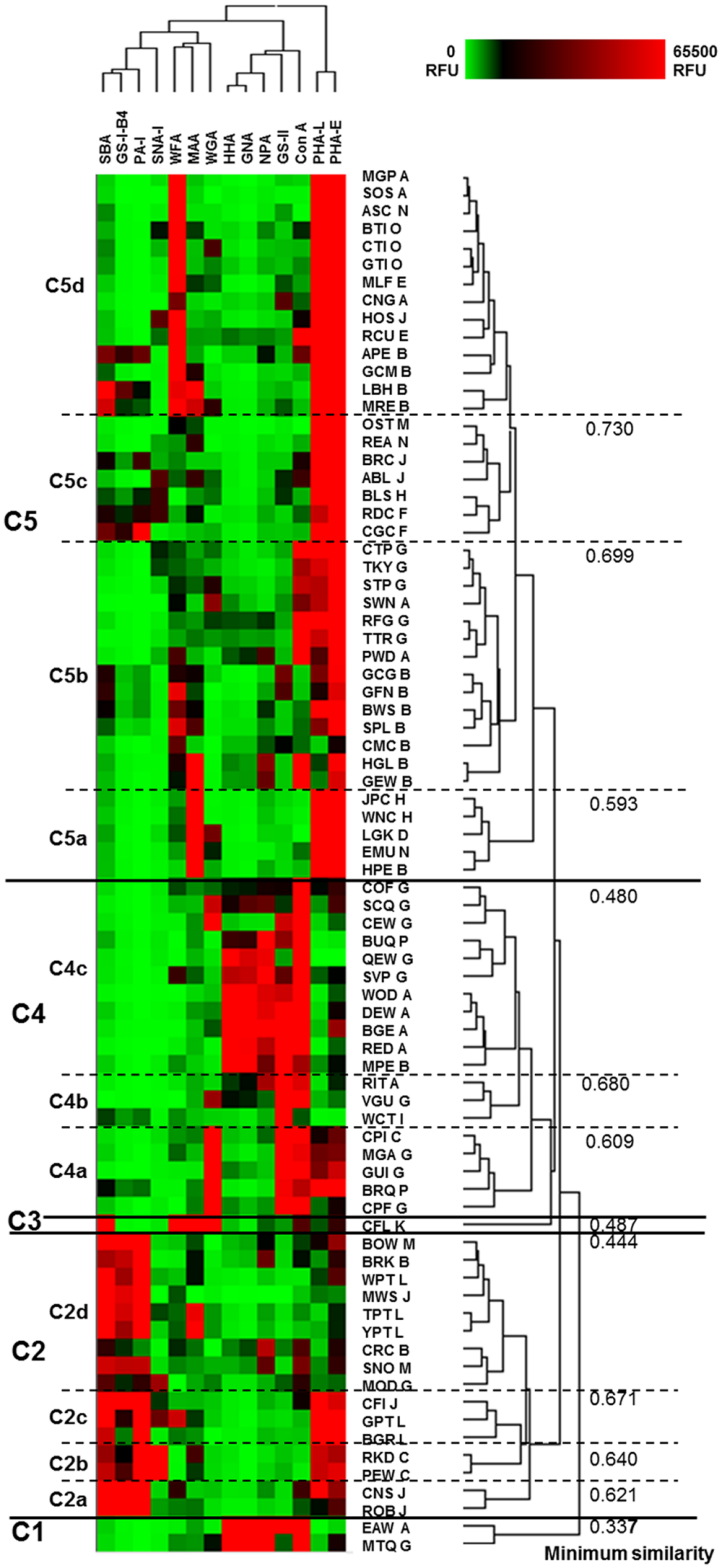
Clustering of the binding interactions of 14 lectins with the 78 EWs. Heat map with dendrograms of hierarchical clustering generated using HCE 3.0. Fluorescence intensities correspond to colour as in the legend. Species names are abbreviated and shown with a capital letter denoting the systematic order as in Table S-1. Five clusters of birds (C1-C5) are indicated by solid lines based on the minimum similarity by glycoprofile located at position 0.5 for major clusters and 0.75 for subclusters (broken lines).

There was considerable variation in lectin binding patterns across the 78 avian species EWs examined (Supplementary Fig. S3). The binding intensity data for the 14 lectin library were subjected to hierarchical clustering to identify any similarities within the diverse species (Fig. 3). To facilitate the description of the groups, the clustered heat map was further divided according to the dendrogram generated by the clustering algorithm to yield five clusters of EWs (Fig. 3, clusters 1-5, C1-5). For C1-5, a minimum similarity of 50% was selected as the defining threshold (bar located at position 0.5, Fig. 3) while 75% similarity was selected for subclusters a, b, c and d.

C1 (Fig. 3) included only two species, EAW and MTQ (Supplementary Table S1) from the orders Anseriformes (A) and Galliformes (G), respectively, and was very different from all other samples, only having 33.7% minimum similarity to the rest of the EW glycoprofiles. EWs in C1 exhibited high binding with the Man-specific lectins HHA, GNA, NPA and Con A, and the GlcNAc-specific GSL-II (but not WGA) (Table 1). EWs in this cluster also exhibited an almost total lack of binding with PHA-L and very low interaction with PHA-E. This lectin profile correlates well with the high Man *N*-linked structures previously reported for birds belonging to Anseriformes and Galliformes (Supplementary Fig. S-4)^35^.

C2 (Fig. 3) was grouped into subclusters a-c and included EWs with high binding intensity to galactose-(Gal-)specific lectins soybean agglutinin (SBA, *Glycine max* agglutinin), *Griffonia simplicifolia* lectin I isolectin B4 (GS-I-B4) and *Pseudomonas aeruginosa* lectin I (PA-I) (Table 1) and was mostly devoid of Man-specific lectin binding signals. PHA-L and PHA-E bound intensely to EWs in C2a-c with low binding for EWs in C2d. C3 (Fig. 3) contained only one EW, which was the only representative from Piciformes in this study. CFL EW had high binding intensity with SBA (terminal *N*-acetylgalactosamine, GalNAc), WFA (GalNAc/sulfated GalNAc), MAA, WGA and moderate interaction with Con A but not with GSL-II, which typically indicates a lower incidence of terminal GlcNAc residues, and PHA-E, which taken together indicated the presence of complex, biantennary *N*-linked structures with terminal GalNAc and α-(2,3)-linked sialylation (Table 1).

In contrast to C2, C4a (Fig. 3) consisted of EWs with high binding intensity to GlcNAc-specific lectins GSL-II and WGA and Con A. This may be indicative of a population of hybrid structures or multi-antennary structures terminating with terminal GlcNAc polymers (polyGlcNAc residues) (Fig. 3). Interestingly, the overall lectin profiles of EWs from C1 had similarities with those of C4c, with some additional binding interactions in C4c (Fig. 3), the latter of which included a further five representatives of Galliformes and three Anseriformes. This pattern suggested that the *N*-linked EW structures in C4c were of the high-mannose and hybrid type. C4b and C4a have either much lower (C4b), or lack (C4a), interactions with HHA, GNA and NPA. Among the members of C4a and C4c which demonstrated intense GSL-II binding, representatives of Anseriformes but not Galliformes corresponded well with the abundant GlcNAc previously reported by MALDI-TOF MS in Anseriformes (Figs S-4 and S-5)^35^. Additionally, the five species in C4a showed very high binding with WGA and moderate binding to PHA-L and PHA-E.

High binding intensity with complex structure-specific PHA-E was observed for more than 60% of the EWs in C5. C5 EWs also had high binding intensity with PHA-L, though to a somewhat lesser extent than PHA-E, with lowest PHA-L binding intensity in C4b. The majority of EWs with high binding intensities for PHA-E and PHA-L were in C5, the largest cluster (Fig. 3). C5 subclusters could be further characterised by additional binding to (i) MAA only (C5a), (ii) Con A with high binding intensity, no interaction with PA-I and GS-I-B4, and some moderate binding intensity with Man-specific lectins (C5b), (iii) Gal- and GalNAc-specific lectins (SBA, *Wisteria floribunda* agglutinin (WFA), GS-I-B4, PA-I) and MAA with diverse binding intensities (C5c), and (iv) WFA with high binding intensity (C5d). Only 8 out of 40 EWs in C5 interacted with SNA-I, which is specific for α-(2,6)-linked sialic acid, with no SNA-I binding at all observed in Anseriformes (A) EWs which clustered in C5. Additionally, among the EWs in C5b with high WFA binding intensity, three representatives of Anseriformes were present but none of Galliformes. WFA binding for these three Anseriformes EWs, MGP, SOS and CNG, corresponded well with the low abundance of GalNAc residues in the *N*-linked oligosaccharide structures previously described for Galliformes (Supplementary Fig. S4)^35^.

Overall, most EW samples exhibited high relative binding intensity with PHA-E and PHA-L, which suggested the presence of bi-, tri- and tetra-antennary *N*-linked structures, and the majority of EWs also interacted with Con A. Con A binding to EW glycoproteins was previously demonstrated using lectin blotting which included 75 EW species overlapping with this study^16^. When comparing data from the present study with all overlapping species^16^, similar trends were reported with the majority of EWs interacting with Con A (93% here compared to 84% previously reported) (Fig. S-7). A similar proportion of GS-I binding to EWs was also shown (40% of the species) despite the wider binding specificity of GS-I (Gal/GalNAc specificity) compared to the more narrowly specific GS-I-B4 (exclusively terminal α-linked Gal) used here (Table 1).

Due to the potential for high yields, avian eggs are also a desirable target for producing recombinant therapeutic glycoproteins^36^. Because the major egg allergens for humans are egg glycoproteins, the possibility for inadvertent co-purification of egg allergens exists. Recent studies using glycosylated variants of chicken OVM and OVA showed that allergy response was mainly related to carbohydrate structures and their location^37, 38^. Thus, production of recombinant proteins with desired glycosylation could be achieved by selection of the avian species with desired glycosylation without having to extensively genetically re-engineer birds.

### EW sialic acid linkage specificity

The overall linkage specificity of the EW library sialylation was addressed in this study using MAA and SNA-I lectins for the first time. Overall, binding to MAA was more frequent across samples (Fig. 3) and was dispersed throughout C2, C3 and C5, with all the members of C5a interacting strongly, and very low binding intensities observed in C1 and C4. SNA-I interaction was low or absent across all EWs except for two EWs from Columbiformes, PEW and RKD, grouped in C2b, with intense binding and 12 other EWs in C2 and C5 with moderate binding. This suggested that α-(2,6)-linked sialic acid is less common in the 78 EW species included in this study. The average sialic acid concentration was previously reported as four times greater in Galliformes compared to Anseriformes^35^ and the relative binding intensities of MAA and SNA-I lectins demonstrated similar trends in this study (Supplementary Fig. S5).

The binding of influenza virus depends on the specificity of its surface hemagglutinin for the particular linkage of the terminal sialic acid on lung epithelial cell surface oligosaccharides. This linkage tropism dictates the species specificity of influenza strains as avian influenza virus favours binding to α-(2,3)-linked sialic acid which is found in the upper respiratory tract of birds and human influenza virus binds to α-(2,6)-linked sialic acid found in the human upper respiratory tract^29^. Many vaccines, including those against influenza, are grown in chicken eggs and it has been shown that the egg component glycosylation influences the specificity of the produced influenza virus^39^. Thus, it is beneficial to understand species-specific EW glycosylation, including the presence or absence of certain motifs and linkages to produce a vaccine better formulated for human pathogens.

### Correlation of EW glycoprofiles with avian phylogeny

Glycan composition was previously shown to be dependent on the phylogenetic relationship between the species^16, 35^. According to the current classification, modern birds are divided into three taxa, Ratitae, Galloanserae, and Neoaves (Supplementary Fig. S-6)^16^. Based on that classification, the expression of terminal α-linked Gal in the terminal structure Gal-α-(1,4)-Gal^20, 35, 40^ was confined to the species from Neoaves. In a previous assessment of 148 avian species EWs, approximately 67% of EWs were shown to express Gal-α-(1,4)-Gal-containing glycoproteins based on lectin (GS-I) and Western blotting (anti-P1 monoclonal antibody) analysis^16^. In addition, differences in galactosyltransferase (GalT) expression was reported in various tissues of ostrich, chicken and pigeon from the taxa Ratitae, Galloanserae and Neoaves, respectively^41^. The β-(1,4)-GalT, which forms the type II LacNAc structure, was present in all three birds while the α-(1,4)-GalT, which forms the terminal structure Gal-α-(1,4)-Gal, was present in pigeon only^41^.

In this work (Fig. 3, C2), the EWs with high binding intensity to Gal-specific lectins (SBA, GS-I-B4, PA-I) were from closely related orders of Psittaciformes, Strigiformes, Columbiformes, Passeriformes and Ciconiiformes, all members of Neoaves which represent neighbouring branches of the phylogenetic tree based on DNA relationships (Supplementary Fig. S-6). However, Cuculiformes, Gruiformes and Musophagiformes, which are in close phylogenetic proximity to the aforementioned orders, showed low or no binding to PA-I and GS-I-B4. Interestingly, the EWs from Turniciformes, Piciformes and Coraciiformes, despite being the part of Neoaves but phylogenetically more distinct than Cuculiformes, Gruiformes and Musophagiformes, did not interact with PA-I and GS-I-B4 at all. In addition, no EWs from the superorders Ratitae and Galloanserae interacted with lectins PA-I and GS-I-B4, in agreement with a previous report^16^.

Overall, the hierarchically clustered lectin glycoprofiling analysis (Fig. 3) revealed that the EWs from all species included did not group strictly according to phylogeny. However, several glycosylation clusters and subclusters contained multiple members of one order, e.g. C5b was composed of seven members of Ciconiiformes, five members of Galliformes and two members of Anseriformes. While Galliformes and Anseriformes are phylogenetically close, Ciconiiformes is more phylogenetically distant (Supplementary Fig. S6). A high prevalence of EWs from these three orders, together with one representative from Turniciformes, was also found in C4c. Thus, the detailed glycosylation and hierarchical clustering analyses demonstrated that the similarity of EW glycosylation among different orders of birds is not strictly dependent on the phylogenetic relationship between the species. Previous conclusions that glycosylation is related to phylogeny was based on only one carbohydrate structure, terminal Gal-α-(1,4)-Gal^16^.

### HTP screening of EWs for pathogen-binding

**Figure 4:**
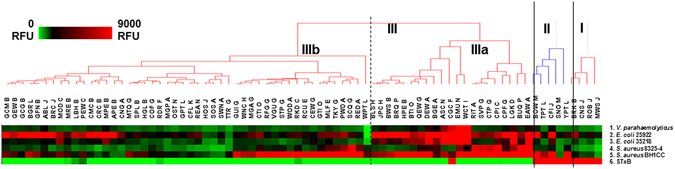
Interactions of the bacteria and Stx-1B with EWs. Heat map with dendrograms of hierarchical clustering generated using HCE 3.0. Fluorescence intensities correspond to colour as in the legend. Species names are abbreviated (Table S-1). Three groups of birds (I–III) are indicated by solid lines based on the minimum similarity by glycoprofile located at position 0.5 for major clusters and 0.75 for subclusters IIIa and IIIb (broken line).

The EW binding of five bacterial strains which can form pili or biofilm was investigated (Table 2). The bacterial strains included were two non-pathogenic *E*. *coli* type strains, the Type 1 fimbriae-producing ATCC 35218 and the P pili-producing ATCC 25922, which bind to Man residues and Gal-α-(1,4)-Gal, respectively, *V*. *parahaemolyticus* RIMD2210633, which is a seafood-borne pathogen known to express type IV pili^42^, and two strains of *S*. *aureus*. *S*. *aureus* is a common food spoilage pathogen^3^ but the strains selected for this study are not food isolates but were selected for their biofilm forming ability. Biofilm formation is a source of persistent contamination in the food processing industry^43^ and persistent infection, for example, in lungs of cystic fibrosis patients or on implanted medical devices. *S*. *aureus* 8325-4 is a methicillin-susceptible isolate which produces a polysaccharide biofilm^44^ while BH1CC is methicillin-resistant and forms a proteinaceous biofilm^45^. Bacterial cells were harvested under conditions optimal for expression of pili or biofilm, fluorescently labelled and incubated on the EW microarrays. The mean binding intensity data was hierarchically clustered (Supplementary Table S-3 and Fig. 4) and three main clusters with 0.5 minimum similarity were identified.

**Table 2:** Bacterial strains and toxins used and their sources.

Strain or toxin	Source or reference
*Escherichia coli* 25922	ATCC
*Escherichia coli* 35218	ATCC
*Vibrio parahaemolyticus* RIMD2210633	O’Boyle, *et al*.^42^
*Staphylococcus aureus* 8325-4	Horsburgh, *et al*.^44^
*Staphylococcus aureus* BH1CC	O’Neill, *et al*.^45^
Cholera toxin subunit B (Ctx-B)	Life Technologies (Carlsbad, CA)
*S*higa-like toxin B (Stx1-B)	Nova Biotech Development (El Cajon, CA)

All selected bacterial strains demonstrated highest relative affinity for three EWs (Supplementary Table S-3), EAW, which interacted with Con A, GS-II and Man-specific lectins, and LGK and EMU, which bound most intensely to complex *N*-linked structure-specific lectins and MAA (Fig. 3). Most EWs which exhibited high intensity interactions with all bacteria were grouped in cluster IIIa and the right- and left- hand side of cluster IIIb, with less interactions in the middle of cluster IIIb (Fig. 4). *S*. *aureus* strains interactions were mainly within the right-hand side portion of cluster IIIb, while the left-hand side of cluster IIIb had higher intensity interactions with the *E*. *coli* strains and moderate binding to the *S*. *aureus* strains.

Four EWs with high Man type structures indicated, EAW, DEW, QEW and BGE, were in the top 15% of most intensely binding EWs for *E*. *coli* ATCC 35218 (Supplementary Table S-3), in agreement with the expected carbohydrate specificity of this strain. For *E*. *coli* ATCC 25922, the top 30% of the most intensely binding EW features included four EWs from the C2 cluster (Fig. 3), CFI, BOW, CRC and BGR, which bound intensely with Gal-specific lectins, including GS-I-B4. This affinity for terminal α-linked Gal was expected for strain ATCC 25922 but interestingly, BRC EW was also among the most intensely bound EWs for this strain. BRC was located in C5c (Fig. 3) and had moderate binding with SBA and PA-I, extremely low binding with GS-I-B4, and high binding with PHA-E and PHA-L. Therefore it is unlikely that BRC has any terminal α-linked Gal but rather terminal β-linked Gal or GalNAc. This observation indicates that strain ATCC 25922 may have multiple adhesins with lectin functionality or that the P pili has wider specificity than Gal-α-(1,4)-Gal alone, which exemplifies the additional advantage of the use of a natural product displaying multiple complex structures as a potential anti-adhesive.

*V*. *parahaemolyticus* demonstrated high binding intensity with WCT EW, which interacted with GSL-II but not with PHA-L and PHA-E. Other intensely *V*. *parahaemolyticus* binding EWs, including EMU, CGC, ASC and LGK, belonged to C5 (Fig. 3) and were bound mainly by PHA-L and PHA-E. The high Man-type structure containing EWs DEW, EAW and BGE, all from Anseriformes, were also found within the top 10% of intensely binding EWs for *V*. *parahaemolyticus*, and binding could potentially be due to the expression of the mannose-sensitive haemagglutinin (MSHA), which has lectin functionality^42^.

To our knowledge, there are no reports indicating lectin functionality of the surface proteins of the *S*. *aureus* strains. However, these strains bound to EWs from clusters C4 and C5, known to bind to i) Con A, GS-II and Man-specific lectins (BUQ, EAW, SCQ, SVP, RED, BGE, QEW and DEW), ii) Con A and GS-II (CPI, RIT, WCT, CPF), iii) Con A, PHA-L and PHA-E (CTP, PWD and TKY), iv) MAA, PHA-L and PHA-E (LGK and EMU), and v) WGA, PHA-L and PHA-E (MLF, CTI and GTI). Thus, these *S*. *aureus* strains may bind to multiple structures including high mannose and complex type with terminal GlcNAc residues and terminal α-(2,3)-linked sialic acids, indicating the presence of several lectins. Interestingly, for strain BH1CC, CFI, YKT and BOW EWs were found within the top 30% of the most intense interactions and can help distinguish BH1CC from the less intense interactions of 83252-4 with these EWs. CFI, YPT and BOW were recognized by the Gal-specific lectins SBA, GS-I-B4 and PA-I and were grouped in C2 (Fig. 3) and cluster II (Fig. 4), so BH1CC may have additional specificity for terminal α- and β-linked Gal structures. However, it is unlikely that the Gal-α-(1,4)-Gal motif was selectively recognised by BH1CC as this strain did not similarly intensely bind to the other seven EWs recognised by subunit B of Stx1 (Stx1-B) (see below for further detail). Nevertheless, further confirmation of specific carbohydrate-mediated binding of *S*. *aureus* adhesins is still required.

The presence of certain glycan structures in EWs of selected avian species suggests their inhibitory role for bird pathogen adhesion. Liu, *et al*.^46^ used pigeon ovalbumin with terminal Gal-α-(1,4)-Gal for detection of uropathogenic bacteria in urine samples. Extracts from edible bird’s nest (regurgitated saliva of male *Collocalia swiftlets*), widely consumed by humans as a delicacy and a naturopathic food, strongly inhibit the hemagglutination of human erythrocytes by human, avian and porcine influenza viruses in a host-range-independent manner^47^, possibly due to the high abundance of sialylated high-antennary *N*-linked oligosaccharides^48^. Because wild birds play an important role in the epidemiology of human-associated zoonoses^49^, an understanding of the mechanisms of their immunity as carriers would be highly beneficial for future developments of disease prevention and treatments.

### Binding of bacterial toxins to the EW microarray

The EW binding of two bacterial toxins was investigated (Table 2). Bacterial cholera toxin subunit B (Ctx-B) did not show any significant interaction with any feature on the EW microarray (Supplementary Table S3), except a GM1 monosialoganglioside (Gal-β-(1,3)-GalNAc-β-(1,4)-[NeuAc-α-(2,3)-]Gal-β-(1,4)-Glc-β-Cer)-containing neoglycoconjugate (GM1-HSA) which was included as a positive control^50^. The lack of Ctx-B binding indicated that the GM1 carbohydrate structure was not present in any of the arrayed EWs.

Stx1-B bound to ten EWs above the stringent five times background threshold^19, 51^ (Fig. 4) including i) four representatives of Passeriformes, CFI, CNS, MWS and ROB EWs, ii) two representatives of Strigiformes, BOW and SNO, iii) two representatives of Psittaciformes, TPT and YPT, iv) one representative from Ciconiiformes, BRK; and v) one from Columbiformes, PEW. The binding of Stx1-B indicated the presence of Gal-α-(1,4)-Gal on the EWs, which is the minimum structure required for Stx1-B binding^17^. Interestingly, all ten EWs were in C2 (Fig. 3), showed high binding intensity with SBA, PA-I and GS-I-B4 and are grouped together as taxonomically close orders (Supplementary Fig. S6).

Previously, PEW was shown to be a rich source of Gal-α-(1,4)-Gal-β-(1,4)-GlcNAc (also known as the P1 antigen) terminating structures^16, 21^. Pigeon ovomucoid, pigeon ovalbumin and whole PEW immobilised on Sepharose gels was used for purification of Stx1 from the crude extracts of *E*. *coli* SLT100^17^. Here, PEW had only moderate binding intensity with Stx1-B and ten other EWs had greater binding intensity. This could be due to a more favourable multivalent presentation of the Gal-α-(1,4)-Gal-containing determinants on the EWs with greater binding intensity for engaging the multiple binding sites of the Stx1-B pentamer. Hence, not only PEW but also the other ten EWs interacting with Stx1-B identified in this study may have potential in the development of alternative and highly efficient methods for neutralisation of toxins and other anti-adhesive strategies.

In conclusion, the complex glycosylation profiles provided in this work demonstrated that the similarity of EW glycosylation among different orders of birds is not strictly dependent on the phylogenetic relationship between the species as previously suggested^16^. The dominating linkages of sialic acid in the EW library were determined for the first time using the lectins MAA and SNA-I. The binding of five bacterial strains which form pili or biofilm was screened and a number of EWs were identified as potential anti-adhesives for these pathogens, along with the potential structures recognised by *S*. *aureus* strains for the first time. Two bacterial toxins were profiled and Stx1 was shown to bind to ten EWs with similar glycosylation with greater intensity than previously identified PEW. Using HTP technology for screening pathogen interactions with the 78 EWs from different avian species could increase understanding of carbohydrate-based anti-bacterial mechanisms of egg white components and species-specific antimicrobial defence. This platform provides a valuable opportunity to promote the exploitation of natural products as anti-adhesives that could be targeted for strain and species specificity in the bid for discovery of alternative antimicrobial agents.

## Methods

### Materials

The lyophilised avian EW library was provided by Prof. Y. C. Lee (Johns Hopkins University, USA) (Supplementary Table S1 and the origin is further detailed in refs 53–55). CEW, QEW and DEW separated from eggs were obtained from local providers (farmers market) and lyophilised. CEW OVA, OVT, OVM, LYZ, and glycoprotein standards transferrin, yeast invertase, ASF, A1AT, AGP and mouse monoclonal anti-6X His IgG-CF640R antibody were from Sigma-Aldrich (Dublin, Ireland). Neoglycoconjugate GM1-HSA was obtained from IsoSep AB (Tullinge, Sweden). TRITC-labelled lectins were from EY Laboratories (San Mateo, CA) or Vector Laboratories, Ltd. (Orton Southgate, United Kingdom). PA-I and WGA were labelled with AlexaFluor® 555 (Life Technologies, USA) according to the manufacturer’s protocol. Cholera toxin subunit B (CT-B) conjugated with AlexaFluor® 555, NuPAGE® Novex® 10% and 4–12% Bis-Tris polyacrylamide gels and SeeBlue® Plus2 pre-stained molecular mass protein standards were purchased from Life Technologies (Carlsbad, CA). Shiga-like toxin B (SLT-1B) with a polyhistidine tag (6x His) attached at its N-terminus was from Nova Biotech Development (El Cajon, CA) (Table 2). The silver stain kit was from Pierce (Thermo Fisher Scientific, Dublin, Ireland). All other materials were from Sigma-Aldrich Co. unless otherwise indicated and were of the highest grade available.

### Sodium dodecyl sulfate-polyacrylamide gel electrophoresis (SDS-PAGE) analysis

EW solutions (Supplementary Table S-1, 5 mg/ml) were prepared in PBS, pH 7.4 and mixed by inversion (4 rpm) at room temperature for 1 h. Samples were first centrifuged for 10 min at 4,000 rpm, the floating layer of aggregate was removed and the sample was centrifuged again for 10 min at 14,000 rpm. Samples were filtered through a 0.2 μm centrifugal filter with PVDF membrane which was washed with PBS before use. The protein content of the final preparation was ascertained using the bicinchonic acid assay. All EW solutions were adjusted to protein concentrations of 1.2 mg/ml in PBS while standards were adjusted to 1 mg/ml in PBS. EWs (1–3 μg per lane) and purified chicken standards (OVA, OVM, OVT and LYZ, 0.5 μg of each) were electrophoresed on NuPAGE® Novex® 10% or 4–12% Bis-Tris polyacrylamide gels under reducing conditions using MOPS buffer at 150 V constant. Protein migration was visualised by silver staining the gels.

### EW microarray construction

Microarray printing was performed essentially as previously described^19, 31^ with minor modifications as follows. Microarrays were printed with a SciFLEXARRAYER S3 (Scienion) piezoelectric printer equipped with a 90 μm glass nozzle with a hydrophobic coating. Crude EWs (probes) were printed onto Nexterion® Slide H microarray slides at 0.6 mg/mL in PBS containing 0.01% Tween 20 and OVA and OVM were printed at 0.5 mg/mL in PBS containing 0.015% Tween 20. Eight identical subarrays were printed per slide, with each subarray containing six replicates each of 52 different probes. The EW library was divided into twinned panels (A and B) for printing, with each panel consisting of EWs from 36 different species of birds and 17 overlapping probes per panel printed at identical locations (Supplementary Table S2). The overlapping probes consisted of six EWs (EAW, PEW, QEW, CEW, DEW, GEW), four CEW glycoproteins (OVA, OVM, OVT and LYZ), six standard glycoproteins (bovine transferrin, yeast invertase, bovine asialofetuin (ASF), bovine fetuin, human α-1-antitrypsin (A1AT) and human α-1-acid glycoprotein (AGP) and one neoglycoconjugate (GM1-BSA).

### Microarray incubation and scanning

For incubations, carried out in triplicate, fluorescently-labelled lectins (Table 1) and toxins (Table 2) were diluted in Tris buffered saline supplemented with divalent cations (TBS; 20 mM Tris-HCl, 100 mM NaCl, 1 mM CaCl_2_, 1 mM MgCl_2_, pH 7.2) with 0.05% Tween 20 (TBS-T). All labelled lectins and toxins were first titrated on the EW microarrays to determine the optimum concentration such that the resulting fluorescence intensity of the bound lectins was below saturation, approximately 65,500 relative fluorescence units (RFU), with minimal background (Table 1). Inhibitions were performed in parallel by diluting lectins in solutions of the haptenic sugar or glycoprotein in TBS-T (Table 1) and pre-incubated for 20 min at room temperature. Microarrays were incubated at 23 °C for 1 h, washed and dried as previously described^19, 31^. After drying, microarray slides were scanned immediately in an Agilent G2505B microarray scanner (532 nm laser, 90% PMT and 5 μm resolution). Images were saved as *.tif files for data extraction. For the His-tagged Stx1-B, the slides were washed and dried after incubation with Stx1-B and then incubated immediately afterwards with AlexaFluor® 647-labelled anti-His antibody (0.5 μg/ml TBS-T). The rest of the procedure was carried out as described above, except using the 633 nm laser for scanning.

### Bacterial culture

Bacterial strains were obtained from ATCC (*E*. *coli* type strains ATCC 35218 and 25922) or as a donation (Table 2). The strains were routinely grown in LB broth and LB agar (*E*. *coli* type strains) or in BHI broth and BHI agar (*V*. *parahaemolyticus* RIMD2210633 and *S*. *aureus* strains 8325-4 and BH1CC). Selected strains were first grown for 16 h (overnight) static at 37 °C in 5 mL liquid culture in a 50 mL plastic tube. Then overnight culture of each strain was inoculated into fresh medium to starting OD_600nm_ 0.05 and grown to mid-exponential phase (OD_600nm_ 0.6). Bacteria were harvested by centrifugation (5,000 × g, 5 min, 20 °C), washed twice in TBS and resuspended in TBS at half of the initial culture volume at an OD_600nm_ of 2.0 (2 × 10^9^ cfu/mL) and 0.5 ml aliquots were prepared.

### Fluorescent staining of bacteria and incubation on EW microarrays

All following steps were carried out with limited light exposure. The optimum concentration of SYTO® 82 fluorescent cell-permeable nucleic acid dye was determined for each strain as previously described^52^. Bacteria aliquots were incubated at 37 °C for 1 h with rotation (200 rpm) with 10 μM SYTO® 82 and then washed seven times in 2 mL of TBS (3,000 rpm, 2 min) each wash to remove excess dye. Finally cells were resuspended in 0.5 mL of TBS with 0.05% Tween-20 (TBS-T) and adjusted to an OD_600nm_ of 1.0 for immediate use on the microarrays. Each labelled strain was titrated to determine optimal dilution and then incubated in triplicate on the EW microarrays as described above.

### Data extraction and analysis

Data extraction and analysis was performed as previously described^19, 31^. Local background-corrected median feature intensity data (F543median-B543) was analysed. The median of six replicate spots per subarray was handled as a single data point for graphical and statistical analysis. Data from the twinned panels and three replicates were normalised across all six microarray slides (three replicates of twinned microarrays) to the per-subarray total intensity mean of the 17 overlapping probes (Supplementary Table S3). Binding data were presented in histogram form of mean intensity with error bars of one standard deviation of all replicates. Unsupervised clustering of the data was also performed using Hierarchical Clustering Explorer v3.0 (HCE 3.0, University of Maryland, http://www.cs.umd.edu/hcil/hce/bec3.html).

### Data availability statement

The authors are willing to make data available upon request.

## Electronic supplementary material

Supplementary information

Supplementary Table S3
