# Supplementary material for: Rapid screening for specific glycosylation and pathogen interactions on a 78 species avian egg white glycoprotein microarray
Source: Sci Rep. 2017 Jul 25;7:6477. doi: 10.1038/s41598-017-06797-6 (PMC5526940; doi:10.1038/s41598-017-06797-6)
Supplement: Supplementary file 1 — Supplementary information [file 41598_2017_6797_MOESM1_ESM.pdf]

Supplementary Information

Rapid screening for specific glycosylation and pathogen interactions on a 78 species avian egg white glycoprotein microarray

Marta Utratna[1], Heidi Annuk[1], Jared Q. Gerlach[1,2], Yuan C. Lee[3], Marian Kane[1], Michelle Kilcoyne[4], Lokesh Joshi[1]

[1]Glycoscience Group, National Centre for Biomedical Engineering Science, National University of Ireland Galway, Galway, Ireland

[2] Regenerative Medicine Institute, National University of Ireland Galway, Galway, Ireland

[3] Department of Biology, Johns Hopkins University, 3400 North Charles Street, Baltimore, Maryland 21218, U.S.A.

[4] Carbohydrate Signalling Group, Microbiology, School of Natural Sciences, National University of Ireland Galway, Galway, Ireland

Table of Contents

LIST OF SUPPLEMENTARY FIGURES AND TABLES S-2

RESULTS AND DISCUSSION S-3

- Solubilisation optimisation S-3
- Optimisation of EW microarray printing S-3
- Performance of lectins with printed glycoprotein standards S-5
- Additional tables S-7
- Additional figures S-11

REFERENCES S-15

LIST OF SUPPLEMENTARY FIGURES AND TABLES

Figure S1. Optimisation of EW's for printing.

Figure S2. Analysis of glycoproteins (other than chicken EW glycoproteins) included as controls in the two panels of EW microarray.

Figure S3. PHA-E lectin binding profile across all EWs.

Figure S4. *N*-linked structures unique for either Anseriformes or Galliformes.

Figure S5. SNA-I and MAA lectin binding profiles of the 28 species representing Anseriformes and Galliformes included in this study.

Figure S6. Phylogenic tree of birds used in this study according to DNA-DNA hybridisation[1] (adapted from Suzuki, *et al.*, 2004[2]).

Table S1. Species of origin of avian EW microarray. Birds grouped by systematic order indicated by an assigned capital letter (A-P).

Table S2. Two printing panels of EW microarray. The samples are listed based on their printing location.

Table S3 (.xls file). Data for lectin and toxin interactions with EW microarray. Normalised fluorescence intensities from incubations of all lectins used in this study, together with their respective inhibitions (**Table 1**) and two toxins with the EW microarray (78 EWs and 17 overlapping standards). Standard deviation for all replicates included. Percentage inhibition was calculated based on the mean values. Coefficient of variation expressed as a percentage (%CV) for all interactions and inhibitions was included.

RESULTS AND DISCUSSION

Optimisation of EW solubilisation

Prior to electrophoresis, solubilisation optimisation was carried out on lyophilised pigeon EW (PEW), gull EW (GEW), CEW, DEW and QEW samples to ensure maximum protein retention. Solubilisation in PBS resulted in a proportion of insoluble aggregates which were removed by centrifugation and filtration. Subsequent delipidisation and acetone precipitation resulted in significant loss of protein, e.g. the protein content of GEW and PEW decreased to 20% and 24% of the original PBS solubilised content, respectively, after delipidisation and 9% and 18%, respectively, after acetone precipitation (data not shown). Fresh CEW is approximately 12% protein by weight with only approximately 0.2% lipid and 0.7% free carbohydrate content,[3] and centrifugation and filtration was found to be sufficient prior to SDS-PAGE analysis and microarray printing.

Optimisation of EW microarray printing

The Nexterion® Slide H microarray slides were selected for optimal background and chemical compatibility as previously described.[4,5] PEW, GEW, CEW, DEW and QEW samples were used to optimise microarray printing and were printed at 0.1 to 0.6 mg/ml, either in PBS or PBS with 0.05% Tween 20 (PBS-T) using a piezoelectric dispenser capillary (PDC) with a hydrophobic coating. The printed slides were incubated with a panel of selected TRITC-labelled lectins (**Table 1**) to monitor the printed feature quality. The lectins were selected based on the glycosylation motifs expected to be present in the EW samples.[2,6] Not all interactions were inhibitable (Supplementary **Table S3** and **Figure S2**). The average inter-array coefficient of variance (%CV) for all lectins was ~16% (range 5% to 45%) for the 78 EWs and ~16% (range <1% to 40%) for the glycoprotein standards (Supplementary **Table S3**). These ranges were lower than expected based on the high viscosity of the samples and %CV reported for other protein-based microarrays.[4,7]

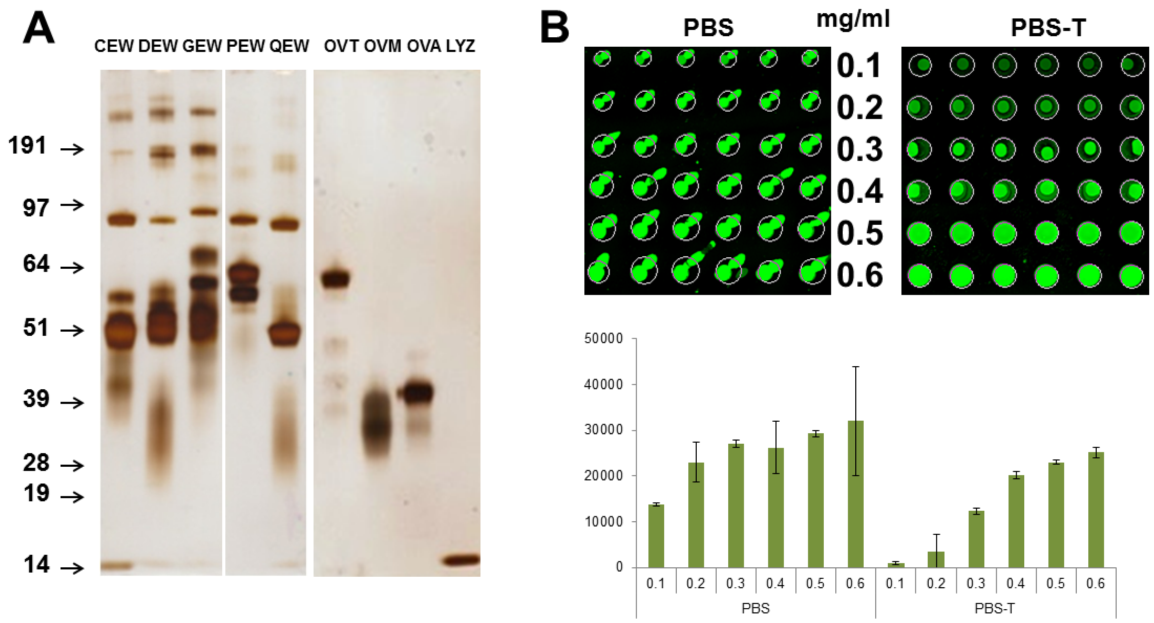

Figure S1. Optimisation of EWs for printing. A) Protein profiles of selected EWs. Samples of five EWs (1 µg per lane, dissolved in PBS pH 7.4) were separated on 10% SDS-PAGE /MOPS and silver stained; commercial (Sigma) purified chicken standards (0.5 µg per lane): ovotransferin (OVT), ovomucoid (OVM), ovalbumin (OVA) and lysosyme (LYZ) were used. B) Schematic of printing layout for the PEW sample printed in gradually increasing concentrations: 0.1-0.6 mg/mL in PBS pH 7.4 (left half) or 0.05% PBS-T (right half). Each sample was printed six times, resulting in six replicate spots. The example illustrates the binding of PEW with PHA-E incubated at a concentration of 0.7 µg/ml. An extraction grid was loaded on top of the imaged spots to indicate the challenges of data extraction, including proper feature fitting. C) Histogram illustrating the mean fluorescence intensity from part B to compare the variability within six replicates. Error bars indicate the standard deviation for the fluorescence intensity of six replicate features for each sample.

Five lectins from the initial library (VVA-B4, PNA, VRA, UEA-I and Jacalin) demonstrated very low binding (data not shown) and did not reach the threshold for inhibition (a minimum of 25% reduction of binding intensity upon co-incubation with appropriate hapten on average for all printed samples). Thus, these five lectins were excluded from the subsequent glycosylation analysis which included the remaining 14 lectins (**Table 1**).

Uniform feature morphology is required for reliable microarray data extraction and the addition of detergent resulted in round features with an average size of 150 μm (Supplementary **Figure S1B**). A concentration-dependent increase in lectin binding was observed up to 0.6 mg/ml, the maximum concentration possible to print for the majority of EWs due to their limited availability (Supplementary **Figure S1C**). Standard glycoproteins were included to monitor lectin performance and were printed at 0.5 mg/ml with 0.01% Tween 20 with the exception of OVA and OVM, which were optimally printed with 0.015% Tween 20 (Supplementary **Table S2**).

Performance of lectins with printed glycoprotein standards

Glycoprotein standards printed on the EW microarray (Supplementary **Figure S2**) demonstrated the expected associations with lectins including binding of the mannose- (Man-) specific lectins NPA and GNA to yeast invertase, which has high- and oligo-mannose type *N*-linked oligosaccharides.[7] The absence of MAA binding to asialofetuin (ASF) confirmed the loss of sialic acid when compared to fetuin and MAA also bound to the serum globulins human α-1-antitrypsin (A1AT), human α-1-acid glycoprotein (AGP) and bovine transferrin, as previously reported).[4]

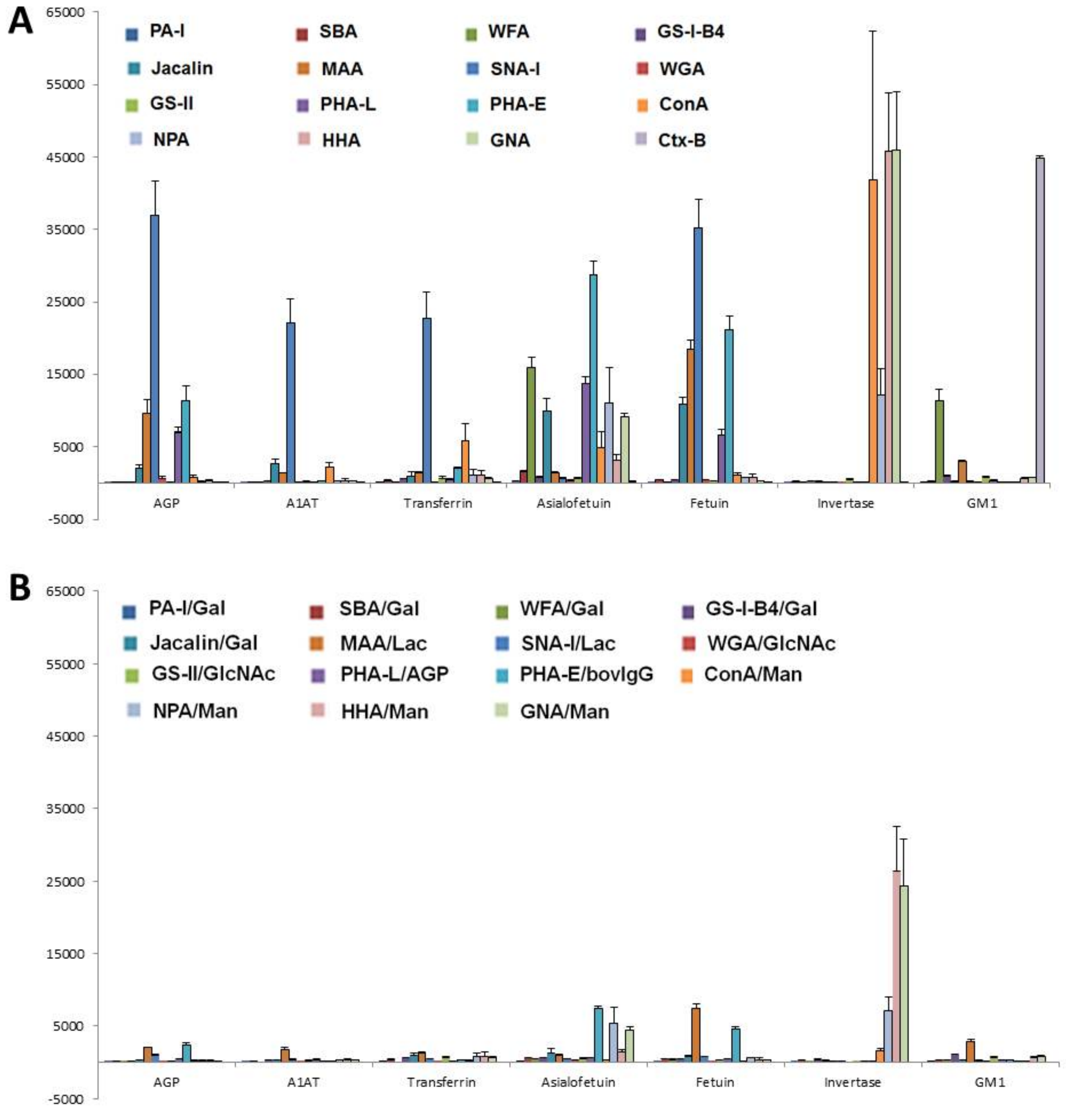

Figure S2. Analysis of glycoproteins (other than chicken EW glycoproteins) included as controls in the two panels of EW microarray. A) Lectin profiles, B) inhibition. Lectin binding profiles of fifteen selected lectins recognised as specific interaction and cholera toxin (Ctx-B), together with their concentrations listed in brackets. The lectin AIA (Jacalin) had carbohydrate inhibitable binding for the glycoprotein standards (non-EW) included on the microarray as controls (included in this figure in the carbohydrate-mediated lectin binding library). The fluorescence intensity values shown are the average of the normalised data from three replicates, with error bars indicating one standard deviation. Error bars are one standard deviation for the mean of all replicates (Supplementary **Table S3**).

Additional tables

Table S1. Species of origin of avian EW microarray. Birds grouped by systematic order indicated by an assigned capital letter (A-P).

Common name	Genus and species	Abbreviation	Order code	Order	Family
Magpie goose	*Anseranas semipolmata*	MGP	**A**	Anseriformes	Anseranatidae
Plumed whistling-duck	*Dendrocygna eytoni*	PWD	**A**	Anseriformes	Dendrocygnidae
Redhead	*Aythya americana*	RED	**A**	Anseriformes	Anatidae
Ringed teal	*Callonetta (Anas) leucophrys*	RIT	**A**	Anseriformes	Anatidae
Barrow's goldeneye	*Bucephala islandica*	BGE	**A**	Anseriformes	Anatidae
Eurasian widgeon	*Anas penelope*	EAW	**A**	Anseriformes	Anatidae
Domesticated duck	*Anas platyrhynchos*	DEW	**A**	Anseriformes	Anatidae
Wood duck	*Aix sponsa*	WOD	**A**	Anseriformes	Anatidae
Black swan	*Cygnus atratus*	SWN	**A**	Anseriformes	Anatidae
Canada goose	*Branta canadensis*	CNG	**A**	Anseriformes	Anatidae
Southern screamer	*Chauna torquata*	SOS	**A**	Anseriformes	Anhimidae
Gull	*Larus argentatus*	GEW	**B**	Ciconiiformes	Laridae
Herring gull	*Larus argentatus*	HGL	**B**	Ciconiiformes	Laridae
Black-winged stilt	*Himantopus himantopus*	BWS	**B**	Ciconiiformes	Charadriidae
Spur-winged lapwing	*Vanellus spinosus*	SPL	**B**	Ciconiiformes	Charadriidae
Adelie penguin	*Pygoscelis adeliae*	APE	**B**	Ciconiiformes	Spheniscidae
Humboldt penguin	*Spheniscus humboldti*	HPE	**B**	Ciconiiformes	Spheniscidae
Macaroni penguin	*Eudyptes chrysolophus*	MPE	**B**	Ciconiiformes	Spheniscidae
Brahminy kite	*Haliastur indus*	BRK	**B**	Ciconiiformes	Accipitridae
Crested caracara	*Polyborus plancus*	CRC	**B**	Ciconiiformes	Falconidae
Chimango caracara	*Milvago chimango*	CMC	**B**	Ciconiiformes	Falconidae
Great-crestec grebe	*Podiceps cristatus*	GCG	**B**	Ciconiiformes	Podicipedidae
Great cormorant	*Phalacrocorax carbo*	GCM	**B**	Ciconiiformes	Phalacrocoracidae
Greater flamingo	*Phoenicopterus ruber*	GFN	**B**	Ciconiiformes	Phoenicopteridae

Mascarene reef-egret	*Egretta dimorpha*	MRE	**B**	Ciconiiformes	Ardeidae
Little blue heron	*Egretta caerulae*	LBH	**B**	Ciconiiformes	Ardeidae
Domestic pigeon	*Columba liviadomestica*	PEW	**C**	Columbiformes	Columbidae
Common crowned pigeon	*Goura cristata*	CPI	**C**	Columbiformes	Columbidae
Mourning dove	*Zenaida macroura*	MOD	**C**	Columbiformes	Columbidae
Rock dove	*Columba livia*	RKD	**C**	Columbiformes	Columbidae
Laughing kookaburra	*Dacelo novaeguineae*	LGK	**D**	Coraciiformes	Halcyonidae
Malle fowl	*Leipoa ocellata*	MLF	**E**	Craciformes	Megapodiidae
Razor-billed curassow	*Crax mitu*	RCU	**E**	Craciformes	Cracidae
Coral-billedground-cuckoo	*Carpococcyx renauldi*	CGC	**F**	Cuculiformes	Cuculidae
Roadrunner	*Geococcyx californianus*	RDR	**F**	Cuculiformes	Neomorphidae
Domesticated chicken	*Gallus gallus domesticus*	CEW	**G**	Galliformes	Phasianidae
Wild turkey	*Meleagris gallopavo*	TKY	**G**	Galliformes	Phasianidae
Coqui francolin	*Francolinus coqui*	COF	**G**	Galliformes	Phasianidae
Scaled (blue) quail	*Callipepla squamata pallida*	SCQ	**G**	Galliformes	Odontophoridae
Japanese quail	*Coturnix japonica*	QEW	**G**	Galliformes	Phasianidae
Mountain quail	*Oreortyx pictus*	MTQ	**G**	Galliformes	Odontophoridae
Congo peafowl	*Afropavo congensis*	CPF	**G**	Galliformes	Phasianidae
Cabot's tragopan	*Tragopan caboti*	CTP	**G**	Galliformes	Phasianidae
White-tailed ptarmigan	*Lagopus leucurus*	TTR	**G**	Galliformes	Phasianidae
Malay great argus	*Argusianus argus*	MGA	**G**	Galliformes	Phasianidae
Ruffed grouse	*Bonasa umbellus*	RFG	**G**	Galliformes	Phasianidae
Satyr tragopan	*Tragopan Satyra*	STP	**G**	Galliformes	Phasianidae
Silver pheasant	*Lophura nycthermera*	SVP	**G**	Galliformes	Phasianidae
Helmet guineafowl	*Numida Meleagris*	GUI	**G**	Galliformes	Numididae
Vulturine guineafowl	*Acryllium vulturinum*	VGU	**G**	Galliformes	Numididae
Black-legged seriema	*Chunga burmeisteri*	BLS	**H**	Gruiformes	Cariamidae
Japanese crane	*Grus japonensis*	JPC	**H**	Gruiformes	Gruidae

White-naped crane	*Grus vipio*	WNC	**H**	Gruiformes	Gruidae
White-cheeked turaco	*Turaco leucotis*	WCT	**I**	Musophagiformes	Musophagidae
Albert's lyrebird	*Menura alberti*	ABL	**J**	Passeriformes	Menuridae
Blue-shouldered robin-chat	*Cossypha cyanocampter*	BRC	**J**	Passeriformes	Muscicapidae
Crimson finch	*Neochmia phaeton*	CFI	**J**	Passeriformes	Passeridae
House sparrow	*Passer domesticus*	HOS	**J**	Passeriformes	Passeridae
Common starling	*Sturnus vulgaris*	CNS	**J**	Passeriformes	Sturnidae
Masked woodswallow	*Artamus personatus*	MWS	**J**	Passeriformes	Corvidae
American robin	*Turdus migratorius*	ROB	**J**	Passeriformes	Muscicapidae
Common flicker	*Colaptes auratus*	CFL	**K**	Piciformes	Picidae
Budgerigar	*Melopsittacus undulatus*	BGR	**L**	Psittaciformes	Psittacidae
Grey parrot	*Psittacus erithacus*	GPT	**L**	Psittaciformes	Psittacidae
Turquois-fronted parrot	*Amazona aestiva*	TPT	**L**	Psittaciformes	Psittacidae
White-capped parrot	*Pionus senilis*	WPT	**L**	Psittaciformes	Psittacidae
Yellow-headed parrot	*Amazoa ochrocephala*	YPT	**L**	Psittaciformes	Psittacidae
Barn owl	*Tyto alba*	BOW	**M**	Strigiformes	Tytonidae
Snowy owl	*Nyctea scandiaca*	SNO	**M**	Strigiformes	Strigidae
Australian cassowary	*Casuarius casuarius*	ASC	**N**	Struthioniformes	Casuariidae
Emu	*Dromaius novaehollandiae*	EMU	**N**	Struthioniformes	Casuariidae
Ostrich	*Struthio camelus*	OST	**N**	Struthioniformes	Struthionidae
Greater rhea	*Rhea americana*	REA	**N**	Struthioniformes	Rheidae
Brushland tinamou	*Nothoprocta cinerascens*	BTI	**O**	Tinamiformes	Tinamidae
Elegant crested-tinamou	*Euotromia elegans*	CTI	**O**	Tinamiformes	Tinamidae
Great tinamou	*Tinamus major*	GTI	**O**	Tinamiformes	Tinamidae
Brown-rumped buttonquail	*Turnix nana*	BRQ	**P**	Turniciformes	Turnicidae
Small buttonquail	*Turnix sylvatica*	BUQ	**P**	Turniciformes	Turnicidae

Table S2. Two printing panels of EW microarray. The samples are listed based on their printing location.

PANEL_A				PANEL_B			
	Abbreviation	printing conc. mg/ml	final Tween20	Abbreviation	printing conc. mg/ml	final Tween20	Sample type:
1	APE (02)	0.6	0.01 %T	ABL (01)	0.6	0.01 %T	Egg white - small scale
2	BGE (05)	0.6	0.01 %T	ASC (03)	0.6	0.01 %T	Egg white - small scale
3	EAW (28)	0.6	0.01 %T	EAW (28)	0.6	0.01 %T	Egg white - small scale
4	BRK (10)	0.6	0.01 %T	BGR (06)	0.6	0.01 %T	Egg white - small scale
5	BTI (12)	0.6	0.01 %T	BLS (07)	0.6	0.01 %T	Egg white - small scale
6	OVA 0.5	0.5	0.015 %T	OVA 0.5	0.5	**0.015 %T**	glycoprotein (chicken egg white)
7	BUQ (13)	0.6	0.01 %T	BOW (08)	0.6	0.01 %T	Egg white - small scale
8	CFL (17)	0.6	0.01 %T	BRC (09)	0.6	0.01 %T	Egg white - small scale
9	OVT 0.5	0.5	0.01 %T	OVT 0.5	0.5	0.01 %T	glycoprotein (chicken egg white)
10	CNG (20)	0.6	0.01 %T	BRQ (11)	0.6	0.01 %T	Egg white - small scale
11	CNS (21)	0.6	0.01 %T	BWS (15)	0.6	0.01 %T	Egg white - small scale
12	OVM 0.5	0.5	0.015 %T	OVM 0.5	0.5	**0.015 %T**	glycoprotein (chicken egg white)
13	COF (22)	0.6	0.01 %T	CFI (16)	0.6	0.01 %T	Egg white - small scale
14	CPI (24)	0.6	0.01 %T	CGC (18)	0.6	0.01 %T	Egg white - small scale
15	Transferrin/Invertase	0.5	0.01 %T	Transferrin/Invertase	0.5	0.01 %T	glycoprotein
16	CTI (26)	0.6	0.01 %T	CMC (19)	0.6	0.01 %T	Egg white - small scale
17	CTP (27)	0.6	0.01 %T	CPF (23)	0.6	0.01 %T	Egg white - small scale
18	ASF	0.5	0.01 %T	ASF	0.5	0.01 %T	glycoprotein
19	GPT (33)	0.6	0.01 %T	CRC (25)	0.6	0.01 %T	Egg white - small scale
20	GTI (34)	0.6	0.01 %T	EMU (29)	0.6	0.01 %T	Egg white - small scale
21	Fetuin	0.5	0.01 %T	Fetuin	0.5	0.01 %T	glycoprotein
22	GUI (35)	0.6	0.01 %T	GCG (30)	0.6	0.01 %T	Egg white - small scale
23	HGL (36)	0.6	0.01 %T	GCM (31)	0.6	0.01 %T	Egg white - small scale
24	PEW	0.6	0.01 %T	PEW	0.6	0.01 %T	Egg white - large scale
25	HOS (37)	0.6	0.01 %T	GFN (32)	0.6	0.01 %T	Egg white - small scale
26	MGA (42)	0.6	0.01 %T	HPE (38)	0.6	0.01 %T	Egg white - small scale
27	GEW	0.6	0.01 %T	GEW	0.6	0.01 %T	Egg white - large scale
28	MGP (46)	0.6	0.01 %T	JPC (39)	0.6	0.01 %T	Egg white - small scale
29	MTQ (48)	0.6	0.01 %T	LBH (40)	0.6	0.01 %T	Egg white - small scale
30	PWD (51)	0.6	0.01 %T	LGK (41)	0.6	0.01 %T	Egg white - small scale
31	QEW	0.6	0.01 %T	QEW	0.6	0.01 %T	Egg white - large scale
32	RCU (52)	0.6	0.01 %T	MLF (43)	0.6	0.01 %T	Egg white - small scale
33	RDR (53)	0.6	0.01 %T	MOD (44)	0.6	0.01 %T	Egg white - small scale
34	DEW	0.6	0.01 %T	DEW	0.6	0.01 %T	Egg white - large scale
35	RED (55)	0.6	0.01 %T	MPE (45)	0.6	0.01 %T	Egg white - small scale
36	RFG (56)	0.6	0.01 %T	MRE (47)	0.6	0.01 %T	Egg white - small scale
37	CEW	0.6	0.01 %T	CEW	0.6	0.01 %T	Egg white - large scale
38	RIT (57)	0.6	0.01 %T	MWS (49)	0.6	0.01 %T	Egg white - small scale
39	RKD (58)	0.6	0.01 %T	OST (50)	0.6	0.01 %T	Egg white - small scale
40	AGP	0.5	0.01 %T	AGP	0.5	0.01 %T	glycoprotein
41	SCQ (61)	0.6	0.01 %T	REA (54)	0.6	0.01 %T	Egg white - small scale
42	SNO (62)	0.6	0.01 %T	ROB (59)	0.6	0.01 %T	Egg white - small scale
43	A1AT/Lysosyme	0.5	0.01 %T	A1AT/Lysosyme	0.5	0.01 %T	glycoprotein / glycoprotein (chicken egg white)
44	SOS (63)	0.6	0.01 %T	SPL (64)	0.6	0.01 %T	Egg white - small scale
45	STP (65)	0.6	0.01 %T	TKY (68)	0.6	0.01 %T	Egg white - small scale
46	GM1-HSA	0.5	0.01 %T	GM1-HSA	0.5	0.01 %T	neoglycoconjugate
47	SVP (66)	0.6	0.01 %T	TPT (69)	0.6	0.01 %T	Egg white - small scale
48	SWN (67)	0.6	0.01 %T	WCT (74)	0.6	0.01 %T	Egg white - small scale
49	PBS-T			PBS-T			PBS-T
50	TTR (71)	0.6	0.01 %T	WNC (75)	0.6	0.01 %T	Egg white - small scale
51	VGU (72)	0.6	0.01 %T	WPT (77)	0.6	0.01 %T	Egg white - small scale
52	WOD (76)	0.6	0.01 %T	YPT (78)	0.6	0.01 %T	Egg white - small scale

16 probes common to both panels for cross normalisation

Additional figures

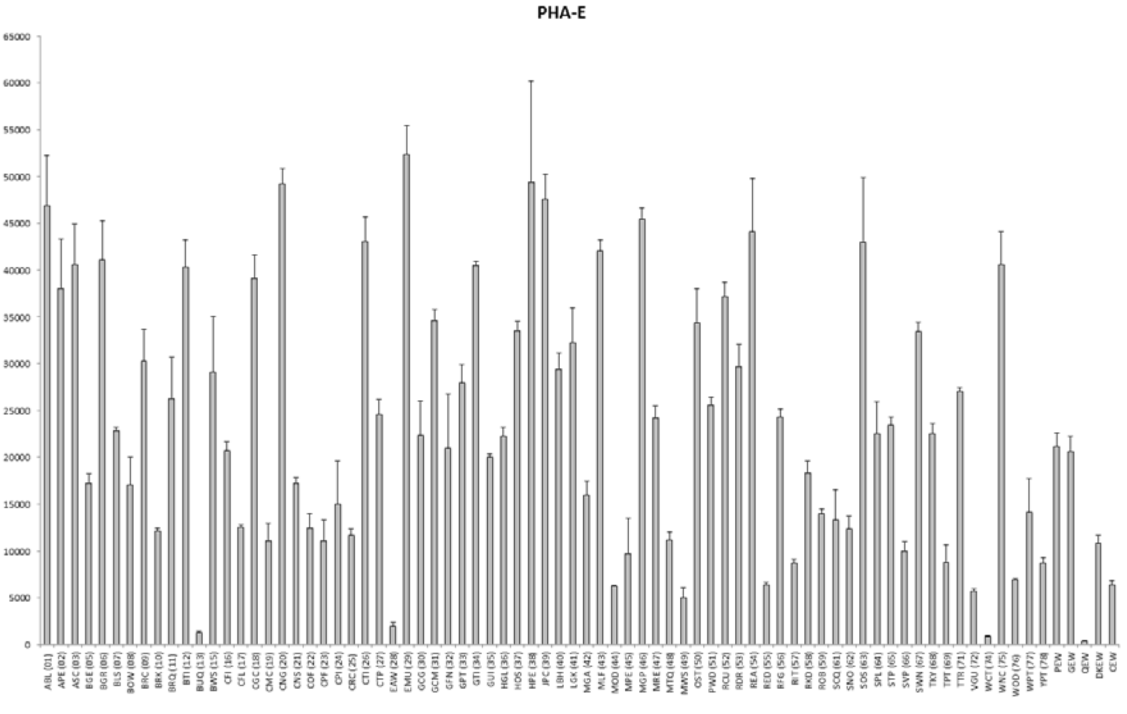

Figure S3. PHA-E lectin binding profile across all EWs. Sample bar chart demonstrating range of binding on the microarray. Error bars are one standard deviation for the mean of all replicates (Supplementary **Table S3**).

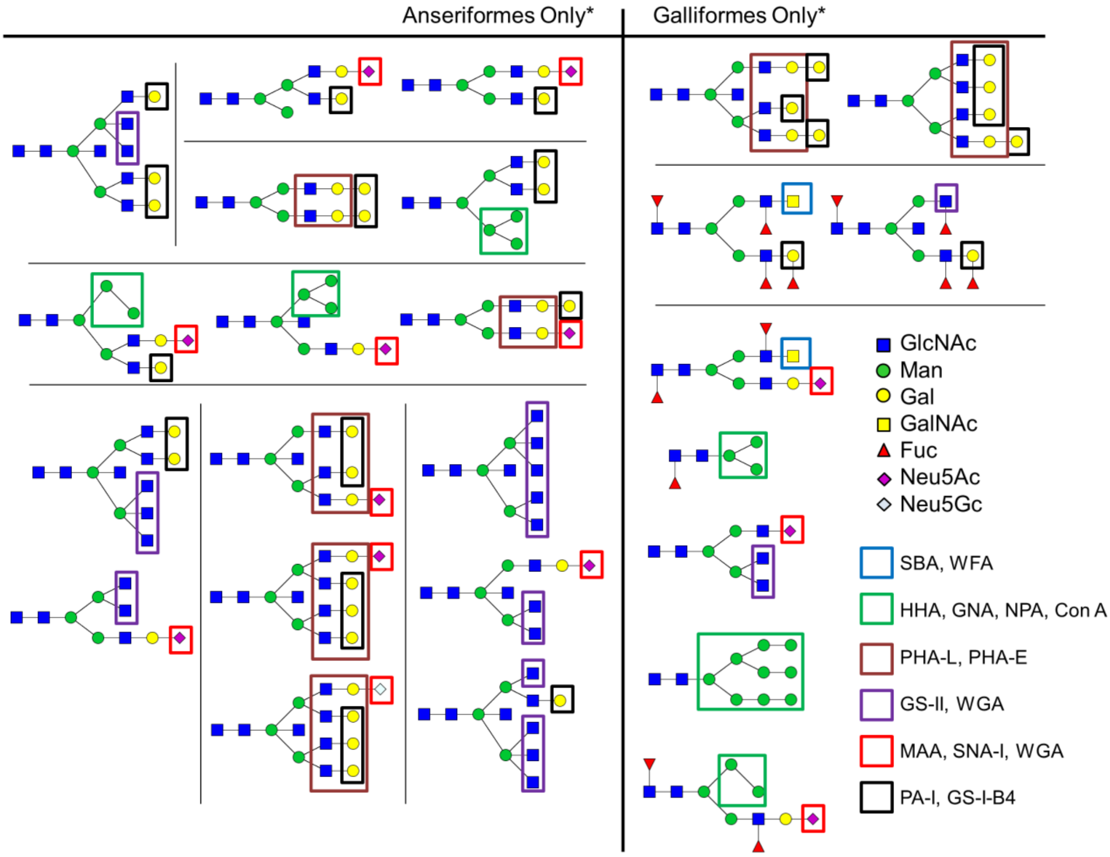

Figure S4. *N*-linked structures unique for either Anseriformes or Galliformes previously reported.[6] Coloured rectangles indicate epitopes for interactions with lectins used in this study (**Table 1**).

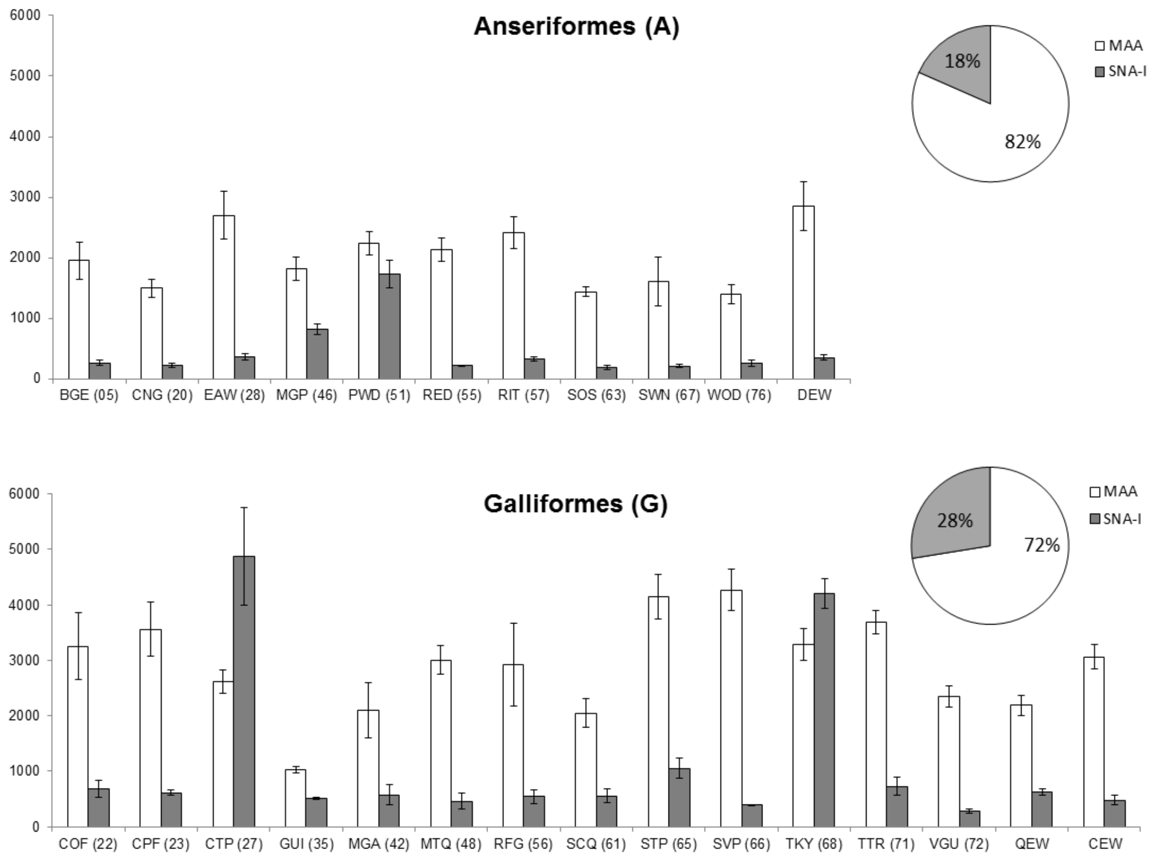

Figure S5. Bar charts for SNA-I and MAA lectin binding to the 28 species representing Anseriformes and Galliformes included in this study. Pie charts indicate proportions of MAA and SNA-I within total intensity of each order. Error bars are one standard deviation for the mean of all replicates (Supplementary **Table S3**).

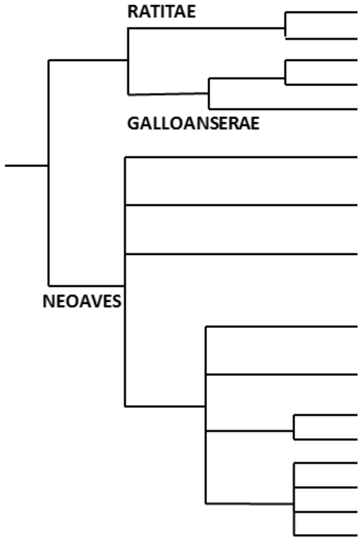

Avian order	Order code	Example	This study: Con A (+)	This study: GS-I-B4 (+)	Overlap	Suzuki *et al.* 2004: Con A (+)	Suzuki *et al.* 2004: GS-I (+)
Struthioniformes	N	ostrich	2	0	4	4	0
Tinamiformes	O	tinamou	3	0	3	3	0
Craciformes	E	currassow	2	0	2	2	0
Galliformes	G	chicken	13	0	13	13	0
Anseriformes	A	duck	9	0	10	9	*0*
Turniciformes	P	buttonquail	2	1	2	2	1
Piciformes	K	flicker	1	0	1	1	0
Coraciformes	D	kookaburra	0	0	1	0	0
Cuculiformes	F	cuckoo	2	2	2	2	2
Psittaciformes	L	parrot	5	5	5	2	5
Musophagiformes	I	turaco	1	1	1	1	1
Strigiformes	M	owl	2	2	2	2	2
Columbiformes	C	pigeon	3	2	3	2	2
Gruiformes	H	crane	2	1	3	2	1
Ciconiiformes	B	heron/gull	14	10	14	10	10
Charadriiformes	J	starling	7	5	7	7	5
		Total:	68	29	73	62	29
			93%	40%		84%	40%

Figure S6. Phylogenic tree of birds used in this study according to DNA-DNA hybridisation[1] (adapted from Suzuki, *et al.*, 2004[2]). The examples of birds were taken from Supplementary **Table S-1** and listed together with a total number of species represented for each order used in this study. The overlap with birds analysed by Suzuki, *et al.*, (2004)[2] is indicated, and the results for Con A and GS-I versus GS-I-B4 are summarised in comparison to this study.

REFERENCES

1. Sibley, C. G. & Monroe Jr, B. L. Taxonomy and distribution of the birds of the world. Yale University Press, New Haven, Connecticut, USA, 1990.

2. Suzuki, N., Laskowski, M. & Lee, Y. C. Phylogenetic expression of Galalpha1-4Gal on avian glycoproteins: glycan differentiation inscribed in the early history of modern birds. *Proc. Natl. Acad. Sci. U. S. A.* **101**, 9023–9028 (2004).

3. Kovacs-Nolan, J., Phillips, M. & Mine, Y. Advances in the value of eggs and egg components for human health. *J. Agric. Food Chem.* **53**, 8421–8431 (2005).

4. Kilcoyne, M. *et al*. Construction of a natural mucin microarray and interrogation for biologically relevant glyco-epitopes. *Anal. Chem.* **84**, 3330–3338 (2012).

5. Kilcoyne, M. e*t al*. *Campylobacter jejuni* strain discrimination and temperature-dependent glycome expression profiling by lectin microarray. *Carbohydr. Res.* **389**, 123–133 (2014).

6. Plasencia, M. D., Isailovic, D., Merenbloom, S. I., Mechref, Y. & Clemmer, D. E. Resolving and sssigning N-linked glycan structural isomers from ovalbumin by IMS-MS. *J. Am. Soc. Mass Spectrom.* **19**, 1706–1715 (2008).

7. Gerlach, J. Q., Kilcoyne, M. & Joshi, L. Microarray evaluation of the effects of lectin and glycoprotein orientation and data filtering on glycoform discrimination. *Anal. Methods* **6**, 440 (2014).
